# Interferential Current Therapy and Spray-And-Stretch in Musculoskeletal Disorders: A Narrative Review of Mechanisms, Evidence, and Clinical Positioning in the Absence of Direct Comparative Trials

**DOI:** 10.3390/bioengineering13101125

**Published:** 2026-09-26

**Authors:** Chul Hee Jung, Seok Yeon Choi, Dong Ha Lee

**Affiliations:** 1 Department of Orthopaedic Surgery, Aerospace Medical Center, Republic of Korea Air Force, Cheongju 28187, Republic of Korea; chulh2006@gmail.com; 2 Department of Rehabilitation Medicine, Aerospace Medical Center, Republic of Korea Air Force, Cheongju 28187, Republic of Korea; csyceo@naver.com; 3 Department of Orthopaedic Surgery, Seoul National University Hospital, 101 Daehak-ro, Jongno-gu, Seoul 03080, Republic of Korea

**Keywords:** electric stimulation therapy, myofascial pain syndromes, trigger points, musculoskeletal pain, cryotherapy, physical therapy modalities, pain management, evidence-based medicine

## Abstract

Background: Interferential current therapy (ICT) and spray-and-stretch are two low-cost, non-invasive modalities that coexist on the treatment menus of outpatient musculoskeletal clinics, yet no published study has directly compared them, and no guidance document provides principled criteria for choosing between them. Objective: To characterise the mechanistic basis, clinical evidence, safety profile, and practical implementation of each modality across the full range of musculoskeletal disorders, and to propose a hypothesis-generating framework for modality positioning in the absence of direct comparative trials. Methods: A narrative review of literature retrieved from PubMed/MEDLINE, Embase, Scopus, the Cochrane Library, PEDro, and Google Scholar from database inception to 1 August 2026, using the search logic (musculoskeletal condition terms) AND (ICT terms OR spray-and-stretch terms), supplemented by hand-searching. The combined search retrieved 3241 records across six databases; 46 primary studies were included after two-reviewer sequential screening. Risk of bias was assessed using the PEDro scale (RCTs) and the Downs and Black checklist (observational studies). No quantitative synthesis was performed. Results: For ICT, multiple randomised trials and meta-analyses across at least four anatomical regions show that stand-alone ICT produces a small and inconsistent analgesic effect versus sham, whereas ICT as a co-intervention yields modest short-term improvements in pain and disability in chronic non-specific low back pain and knee osteoarthritis. For spray-and-stretch, a small number of predominantly fair-quality RCTs, almost all limited to the upper trapezius, show a real but brief effect of the combined procedure; the independent contribution of the vapocoolant has not been isolated in any dismantling study. Conclusions: The two modalities address mechanistically distinct clinical problems and are not directly comparable on the current evidence. We propose a hypothesis-generating positioning framework—unvalidated and intended to guide future comparative trials rather than to serve as a clinical decision rule—in which ICT is considered for deep, regional, multi-session analgesic adjunction, and spray-and-stretch for single-session restoration of range of motion around a discrete taut band. Direct comparative trial data are needed to test this framework.

## 1. Introduction

Musculoskeletal disorders are the leading global cause of years lived with disability. Analyses of the Global Burden of Disease Study 2021 estimate roughly 1.7 billion prevalent cases worldwide, a figure that has nearly doubled since 1990 and is projected to continue rising with population growth and ageing [1,2]. Low back pain alone accounted for 619 million prevalent cases in 2020 [3], and osteoarthritis contributes a further large and expanding share of the total [4]. Myofascial trigger points are identified in a substantial proportion of patients presenting with neck, shoulder, and low back complaints [5,6], and myofascial pain syndrome remains one of the most common yet least standardised diagnoses in musculoskeletal practice [7].

Two low-cost, non-invasive modalities have coexisted on the same outpatient treatment menu for decades without ever being formally compared. Spray-and-stretch combines a rapid topical application of vapocoolant over a muscle with immediate passive lengthening [8]. Interferential current therapy (ICT) delivers two out-of-phase medium-frequency alternating currents through the skin, producing a low-frequency amplitude-modulated envelope intended to engage deeper tissue with less cutaneous discomfort than conventional low-frequency stimulation [9,10].

Both modalities appear in clinical physiotherapy guides for musculoskeletal pain, yet no published study has directly compared them, and no guideline provides principled criteria for choosing between them. Whether clinicians treat them as interchangeable or as distinct tools for distinct presentations is not documented in the literature; this absence of documented criteria is itself the gap this review addresses. A search of ClinicalTrials.gov conducted on 1 August 2026 (terms: “interferential current” AND “spray and stretch”) identified zero registered or completed comparative trials. The closest approximation in the published literature is a single multi-arm trial of cervical myofascial pain modalities that included ICT-based and spray-and-stretch-based arms, but which was not powered for a two-way comparison and embedded the two modalities in different co-interventions [11].

The two modalities differ sufficiently in mechanism, evidence base, and practical application that choosing between them requires a structured rationale—even though no comparative trial exists to validate any such rationale. This narrative review therefore has three aims: to characterise the mechanistic and technical basis of each modality and to make explicit how differently they engage tissue; to appraise the clinical evidence for each across the range of musculoskeletal disorders without conflating two literatures of very unequal size and quality; and to propose a hypothesis-generating framework for positioning them in routine orthopaedic and rehabilitation practice that can serve as the basis for future comparative studies.

## 2. Materials and Methods

This article is a narrative review. It was not prospectively registered. The syntheses presented below represent structured expert appraisal rather than systematic evidence grading.

### 2.1. Search Strategy

PubMed/MEDLINE, Embase, Scopus, the Cochrane Library, PEDro, and Google Scholar were searched from database inception to 1 August 2026. The search logic combined musculoskeletal condition terms with ICT or spray-and-stretch terms using the structure: (Block A) AND (Block B OR Block C), where Block A comprises musculoskeletal terms, Block B comprises ICT terms, and Block C comprises spray-and-stretch terms. This logic retrieves records mentioning musculoskeletal conditions together with either modality and does not require that all three blocks co-occur.

The representative PubMed/MEDLINE search string was: (“musculoskeletal pain”[tiab] OR “myofascial pain”[tiab] OR “trigger point”[tiab] OR “low back pain”[tiab] OR “neck pain”[tiab] OR “shoulder pain”[tiab] OR “osteoarthritis”[tiab]) AND (“interferential current”[tiab] OR “interferential therapy”[tiab] OR “spray and stretch”[tiab] OR “vapocoolant”[tiab] OR “ethyl chloride”[tiab] OR “cold spray”[tiab]). Analogous strings were used for each database. Database-specific yields (search date 1 August 2026): PubMed/MEDLINE, 1084 records; Embase, 891; Scopus, 672; Cochrane Library (CDSR and CENTRAL), 198; PEDro, 143; Google Scholar (first 25 pages of results per concept-pair search), 253; total 3241. Reference lists of retrieved articles, systematic reviews, and relevant clinical guidance documents were hand-searched.

### 2.2. Inclusion and Exclusion Criteria

Inclusion criteria followed a PICOS framework. Population: patients with any musculoskeletal disorder or healthy participants with latent trigger points or post-procedural pain. Intervention: ICT or spray-and-stretch or both. Comparison: sham stimulation or sham spray, no treatment, an active comparator, or between-modality contrast. Outcomes: pain intensity, pressure pain threshold, range of motion, disability indices, or physiological parameters. Study design: randomised controlled trials were prioritised for ICT; non-randomised comparative studies and clinical guidance documents were retained for spray-and-stretch where RCTs were unavailable, and this asymmetry is acknowledged as a limitation. Exclusion criteria: pelvic, visceral, urogynaecological, or purely dermatological applications; animal or in vitro studies; conference abstracts without full-text publication; non-English language reports.

### 2.3. Study Selection

Two authors (C.H.J. and S.Y.C.) independently screened titles and abstracts; disagreements were resolved by discussion with D.H.L. as arbiter. Deduplication was performed in EndNoteX9 before screening; 427 duplicates were identified and removed, yielding 2814 unique records. Google Scholar was screened to a limit of 25 pages per concept-pair search, a pragmatic limit stated explicitly here. Records were screened sequentially: 2814 unique records screened by title and abstract; 312 full-text articles assessed for eligibility; 46 primary studies included in the synthesis. Reasons for exclusion at full-text stage were as follows: wrong population or setting, 71; non-musculoskeletal application, 64; conference abstract only, 52; animal study or in vitro, 44; non-English, 26; duplicate reporting of the same trial cohort, 9. In addition, 10 background and contextual references were identified through hand-searching and are cited in the manuscript but are not treated as primary evidence sources. Of the 56 sources cited in this review, 46 are primary studies included in the evidence synthesis; the remaining 10 are background, contextual, or methodological references.

### 2.4. Risk of Bias Assessment

The PEDro scale was applied to all RCTs included in the ICT synthesis (*n* = 29). The Downs and Black checklist was applied to non-randomised comparative studies and to the spray-and-stretch literature where RCTs were absent or supplemented by observational data. ICT RCTs scored predominantly in the fair-to-good range (PEDro 4–7/10); the main methodological weaknesses were absence of therapist blinding (inherent to the modality), incomplete adverse-event reporting, and short follow-up. Spray-and-stretch trials scored consistently lower (PEDro 3–5/10), primarily due to small samples and inadequate allocation concealment. The characterisations of evidence quality in Section 4 are derived from these study-level appraisals.

## 3. Mechanistic and Technical Basis

Section 3 addresses the mechanistic and technical basis of each modality (Aim 1). Section 4 and Section 5 appraise the clinical evidence for ICT and spray-and-stretch, respectively (Aim 2). Section 6 provides a structured comparative synthesis. Section 7 translates the synthesis into a hypothesis-generating positioning framework (Aim 3).

### 3.1. Interferential Current Therapy

Skin impedance falls steeply as stimulation frequency rises; a medium-frequency alternating current in the kilohertz range therefore crosses the cutaneous barrier far more readily, and with less cutaneous discomfort, than a conventional low-frequency current. Two such currents differing slightly in frequency—for example, 4000 Hz and 4100 Hz—are delivered through separate circuits and intersect within the tissue, generating an amplitude-modulated envelope whose beat frequency equals the difference between the two carriers (in this example, 100 Hz). The resulting low-frequency envelope is intended to depolarise peripheral nerve fibres while retaining the penetration advantage of the medium-frequency carrier [9]. Direct in-tissue dosimetry data in human soft tissue remain limited, and the actual penetration depth in vivo is difficult to verify; claims regarding specific depth of action should be interpreted as design intent rather than confirmed physiological fact.

Two analgesic mechanisms are conventionally invoked. At higher amplitude-modulated frequencies (typically 80–150 Hz), the intention is to recruit large-diameter Aβ afferents and engage segmental gating; at lower frequencies and higher intensities, the intention is to activate descending inhibitory pathways and endogenous opioid release. Experimental work in healthy volunteers has demonstrated hypoalgesic responses to active ICT relative to placebo [12], and immediate analgesic effects have been shown in patients with chronic low back pain in placebo-controlled designs [13]. A randomised comparison of three amplitude-modulated frequencies found improvement across all active arms with differences between frequencies smaller than the active-versus-control difference [14]. Meta-analytic comparison with TENS has found broadly similar analgesic effects, suggesting that the distinctive architecture of ICT may matter less than the fact of comfortable transcutaneous sensory stimulation [15].

### 3.2. Spray-And-Stretch

Spray-and-stretch rests on cutaneous thermal counterirritation. A vapocoolant is applied in unidirectional sweeps along the muscle fibres toward the referral zone; the rapid evaporation produces an abrupt fall in skin temperature. The intended consequence is a transient interruption of the cutaneous afferent barrage and of the reflex resistance to lengthening, creating a brief window during which the clinician can passively take the muscle toward its normal length [8].

The physiological substrate for the cold component is characterised in general terms: cooling slows peripheral nerve conduction in a temperature-dependent manner and raises pain threshold and tolerance [16,17], and controlled measurement has confirmed significant slowing of motor and sensory conduction after local skin temperature reduction [18]. At the extreme end of the same physical spectrum, deliberate nerve freezing produces a durable conduction block—the basis of cryoneurolysis [19]—but a two-to-five-second vapocoolant sweep operates several orders of magnitude below that threshold. The extrapolation from sustained cryotherapy (many minutes of cooling, demonstrable conduction slowing) to a brief vapocoolant sweep (seconds, superficial cooling) is biologically plausible but mechanistically unproven.

The mechanistic asymmetry is important. In ICT, the device is the treatment. In spray-and-stretch, the spray is hypothesised to be a facilitating adjunct; the therapeutic work is hypothesised to be done predominantly by the passive lengthening. This has not been demonstrated in a dismantling study with a stretch-only control arm. Any evaluation of the combined spray-and-stretch procedure that does not include a stretch-only comparator cannot attribute its result to the vapocoolant. Table 1 summarises the technical and mechanistic characteristics of both modalities.

## 4. Interferential Current Therapy in Musculoskeletal Disorders

### 4.1. Evidence by Comparator Type

#### 4.1.1. ICT Versus Sham or Placebo as Stand-Alone Treatment

As a stand-alone intervention, the analgesic effect of ICT versus sham is small and inconsistent. The first large meta-analysis of ICT for musculoskeletal pain reported no significant superiority to placebo or comparison therapies when used alone [10]. A later systematic review with meta-analysis reached a closely similar conclusion: ICT alone was superior to placebo at discharge in a limited number of studies, but the small number of contributing studies constrained confidence [20,21]. For chronic non-specific low back pain specifically, Rampazo et al. (2023) pooled five RCTs (total *n* = 272) comparing stand-alone ICT with sham immediately post-treatment and found a standardised mean difference of −0.89 (95% CI −1.42 to −0.36; I^2^ = 79%) [22]. The authors characterised this as a moderate-to-large effect size, while noting that the high heterogeneity and small number of contributing studies substantially limit confidence. A comparative synthesis of electrical modalities in the same population identified interferential current as among the more effective electrotherapeutic options for patient-reported outcomes [23].

#### 4.1.2. ICT as a Co-Intervention Versus Active Treatment Alone

A more consistent signal emerges when ICT is added to an active programme and compared with that programme alone. In mechanical neck pain, Albornoz-Cabello et al. (2019) randomised 84 patients to exercise alone versus ICT added to exercise and found significant between-group improvements in pain, disability, psychological status, and cervical range of motion favouring the combined arm [24]; a subsequent RCT confirmed immediate clinical benefit of combining exercise and interferential therapy [25]. For knee osteoarthritis, Varapirom et al. (2024) compared ICT plus quadriceps strengthening against sham ICT plus the same exercise programme in a double-blind RCT and isolated a significant ICT increment for pain relief [26,27]. These studies provide the most direct evidence that ICT adds value within an active rehabilitation context. The number of such rigorously designed co-intervention trials remains small.

#### 4.1.3. ICT Versus Active Comparator Treatment

When ICT is compared against an active comparator of similar intensity, evidence of superiority is generally absent. Neither ICT alone nor ICT added to other interventions outperformed active comparator treatments in the Hussein et al. (2022) systematic review [20]. ICT performed comparably to electrical acupuncture in hemiplegic shoulder pain [28,29,30], and adding ICT to exercise and manual therapy for subacromial impingement did not produce incremental benefit. The practical implication is that ICT adds analgesic value in the context of an active rehabilitation programme but does not demonstrably outperform other well-applied active treatments.

### 4.2. Evidence by Anatomical Region

Neck pain: Two RCTs support the incremental benefit of adding ICT to exercise [24,25]. A more recent trial addressing the question of incremental value within an already multimodal programme provides useful context for the limits of that benefit [26]. Electromyographic work has documented upper trapezius relaxation induced by both ICT and TENS in computer users with chronic neck discomfort [27].

Shoulder disorders: Two RCTs found no significant incremental benefit of adding ICT to exercise and manual therapy for subacromial impingement [28,29]. ICT has a plausible role where the dominant problem is regional nociceptive pain rather than tendon loading.

Knee osteoarthritis: Ten RCTs (pooled *n* = 493) showed significant improvement in ICT groups relative to controls for short-term pain (SMD −0.62, 95% CI −1.01 to −0.23), long-term pain, and WOMAC scores [31]. The meta-analysis characterised the evidence as supporting ICT for short-term outcomes [32], with no obvious serious adverse events reported across the included studies. ICT has also been evaluated in juvenile idiopathic arthritis, where combined resistive underwater exercise and ICT improved outcomes in a RCT [33].

### 4.3. Parameters and Dose

The evidence for parameter specificity is weak. Differences between active parameter settings are consistently smaller than the active-versus-control difference [9,14]. The most frequently used configuration is a 4 kHz carrier with an amplitude-modulated frequency of 30–180 Hz applied for 20–30 min at a comfortable intensity. Clinicians should prioritise adequate intensity, adequate session count, and concurrent active exercise over fine adjustment of device settings.

### 4.4. Safety of ICT

Across the randomised trial literature, no serious adverse events attributable to ICT have been reported [10,20,31]. The included studies varied in their systematic monitoring of adverse events, and absence of reported events should not be equated with demonstrated safety unless adverse-event capture was pre-specified; most contributing trials did not report pre-specified adverse-event monitoring. Reported problems are limited to transient cutaneous erythema beneath the electrodes, occasional discomfort at high intensity, and rare contact dermatitis. The established contraindications in electrotherapy device labelling include the following: implanted cardiac devices; application over the pregnant uterus; active malignancy or arterial/venous thrombosis in the treatment field; and impaired sensation or communication in the treatment area [34]. ICT application is often supervised rather than delivered by senior clinicians directly, subject to local scope-of-practice regulations [35].

## 5. Spray-And-Stretch in Musculoskeletal Disorders

### 5.1. Technique

The technique as classically described positions the patient so that the target muscle can be taken passively toward end range, applies the vapocoolant in unidirectional sweeps at roughly 30–60 degrees from 30–45 cm, parallel to the muscle fibres and toward the referral zone, and passively lengthens the muscle concurrently or immediately afterwards. Moist heat and active range-of-motion exercise follow to rewarm the tissue [8].

### 5.2. Clinical Evidence

The evidence base is small and almost entirely confined to the upper trapezius. The most methodologically informative study is a 72 h follow-up single-blind RCT of 70 healthy volunteers with a latent trigger point, in which the combined spray-and-stretch procedure reduced post-needling soreness immediately, but the effect was not maintained beyond six hours [36]. This demonstrates a real, measurable immediate effect of the combined procedure while defining it as short-lived.

A substantial proportion of the spray-and-stretch literature used healthy volunteers with latent rather than active trigger points, or patients with post-procedural soreness rather than primary musculoskeletal disorders. Extrapolation to patients with chronic active trigger points, degenerative spinal disorders, or post-surgical presentations should be made with caution, since the neurophysiological context may differ substantially.

Comparative trials place spray-and-stretch against other manual approaches. An RCT in 60 neck-pain patients with active upper trapezius trigger points found significant between-group differences favouring the integrated neuromuscular inhibition technique over both spray-and-stretch and stretching exercise alone [37]. A comparative interventional study of 70 patients found ischaemic compression and spray-and-stretch both effective for active trapezius trigger points [38]. A prospective RCT in 60 patients found that dry needling and cold-spray-stretching were both effective across multiple outcome measures, and the authors noted that the non-invasive option should not be underestimated [39]. An older study found spray-and-stretch produced measurable change in pain threshold, though it was outperformed by deep pressure soft tissue massage [40].

Three limitations are fundamental. First, sample sizes are small (typically, 60–90 participants). Second, the anatomical scope is narrow: the upper trapezius dominates and generalisation to other regions is unsupported. Third, no study includes a stretch-only control arm; the effect reported in every trial is the effect of the combined spray-and-stretch procedure. Statements that spray-and-stretch “works” are compatible with the stretch doing most or all of the work—a hypothesis that remains formally untested.

### 5.3. Safety

Applied competently, spray-and-stretch is safe; manufacturer labelling records cutaneous sensitisation as extremely rare. Three hazards are specific to this modality and supported by evidence beyond product labelling: (i) over-application produces frostbite and pigmentation change, documented in product Safety Data Sheets and clinical case reports; (ii) ethyl chloride is flammable and must not be used near open flame or electrocautery, per manufacturer labelling; (iii) inhalation produces narcotic and general anaesthetic effects, and deliberate misuse has produced severe and occasionally irreversible neurotoxicity [39], supporting controlled institutional storage. In the RCTs included in this review, adverse-event reporting was generally not pre-specified, and serious events were not reported; the same caveat regarding absent systematic monitoring applies here as for ICT. The passive stretch component carries a small additional risk in patients with hypermobility, recent muscle strain, or post-surgical tissue.

## 6. Comparative Appraisal

The underlying literatures differ by roughly an order of magnitude in volume. ICT has been evaluated in several dozen RCTs and multiple independent meta-analyses across at least four anatomical regions; PEDro scores of included RCTs range predominantly from 4 to 7 (fair to good). Spray-and-stretch has been evaluated in a handful of small RCTs (PEDro 3–5; fair quality), almost entirely in one muscle. Table 2 and Table 3 present the comparative appraisal with this asymmetry in view.

Several contrasts are robust. The temporal profiles differ: ICT accrues benefit over a course of sessions, whereas the combined spray-and-stretch procedure produces an immediate effect that dissipates within hours [41]. The outcome domains differ: ICT trials report pain intensity and disability indices; spray-and-stretch trials centre on range of motion and pressure pain threshold. The evidential weaknesses differ in kind: for ICT, the effect is small and difficult to separate from co-intervention effects; for spray-and-stretch, the effect of the combined procedure is real but brief and cannot be attributed to the vapocoolant independently [42,43,44,45,46,47]. The practical footprints differ: ICT requires a device and a multi-session course; spray-and-stretch can be completed within a single consultation. There is no direct comparative trial [48,49].

**Table 2:** Comparative appraisal of the evidence base.

Evidence Domain	Interferential Current Therapy	Spray-and-Stretch
Volume of RCT evidence	Several dozen RCTs; multiple independent meta-analyses	Small number of RCTs, most *n* < 90; no meta-analysis
Anatomical breadth	Low back, neck, shoulder, knee, paediatric arthritis	Predominantly upper trapezius; minimal evidence elsewhere
Effect vs. sham (stand-alone)	Small and inconsistent; SMD −0.89 (95% CI −1.42 to −0.36; I^2^ = 79%) for NSLBP vs. sham [22]	Not evaluated as stand-alone; combined procedure effect only
Effect as co-intervention	Demonstrated incremental benefit in neck pain [24,25] and knee OA [32]; not demonstrated in shoulder [28,29]	Combined procedure compared against other manual techniques; not isolated from stretch
PEDro scores (RCTs)	Fair to good: 4–7/10; main weaknesses: therapist blinding, adverse-event reporting, follow-up	Fair: 3–5/10; main weaknesses: small *n*, allocation concealment
Duration of demonstrated benefit	Immediately post-treatment to short-term follow-up; long-term data limited	Immediate; shown to dissipate within hours in controlled follow-up [34]
Head-to-head comparison	None identified	None identified

NSLBP, non-specific low back pain; OA, osteoarthritis; SMD, standardised mean difference; CI, confidence interval. Evidence characterisations are derived from study-level PEDro appraisal and cited meta-analyses. The spray-and-stretch “effect” column refers consistently to the combined spray-and-stretch procedure, not to the vapocoolant component independently.

**Table 3:** Safety, practical, and implementation comparison.

Domain	Interferential Current Therapy	Spray-and-Stretch
Common adverse effects	Transient erythema; discomfort at high intensity; rare contact dermatitis	Transient cutaneous cold discomfort; post-stretch soreness
Serious adverse effects	Not reported in RCT literature; adverse-event capture variable across trials	Frostbite and pigmentation with over-application (product labelling and case reports); inhalation neurotoxicity [39]
Agent-specific hazards	None beyond shared electrotherapy contraindications	Flammability of ethyl chloride; inhalation abuse potential [39]
Established contraindications (per device labelling [47])	Implanted cardiac device; pregnant uterus; malignancy/thrombosis in field; impaired sensation	Cold hypersensitivity; anaesthetic or broken skin; unstable or recently repaired musculotendinous unit
Operator requirements	Low-to-moderate; often supervised per local regulations [48]	Moderate-to-high; trigger point identification and stretch control
Portability	Electrotherapy unit and dedicated space required	Fully portable; point-of-care
Time per encounter	20–30 min plus setup	Typically, under 5 min

Adverse-effect frequencies are qualitative summaries; most included studies did not pre-specify adverse-event monitoring protocols.

## 7. A Hypothesis-Generating Framework for Modality Positioning

The following framework lists mechanistic hypotheses to be tested in future comparative trials. No element constitutes a clinical recommendation, and none has been prospectively validated. The phenotypes described are idealised constructs; most patients in clinical practice will have mixed features that do not map cleanly onto a single row.

The two modalities address mechanistically distinct clinical problems and differ sufficiently in mechanism, evidence base, and practical application that choosing between them requires a structured rationale [50]. ICT is designed as an electrophysical agent for regional, multi-session analgesic management. The combined spray-and-stretch procedure is a single-encounter, manual technique for immediate restoration of range of motion in a specific muscle. Their targets, dose structures, and outcome horizons differ. Table 4 proposes hypotheses for modality positioning based on this mechanistic differentiation.

The framework implies attention to what neither modality does well. Where a taut band is genuinely refractory, needle-based and instrument-based interventions have accumulated a larger comparative literature [51,52,53,54,55,56]. These are cited to illustrate that the comparative standard in trigger-point research has risen, and that spray-and-stretch has not yet been evaluated against it.

## 8. Limitations and a Research Agenda

As a narrative synthesis, this review is subject to selection bias in the identification and weighting of studies. Restriction to English-language publications may have excluded relevant regional literature, particularly for spray-and-stretch, a technique with long-established use in several national physiotherapy traditions. The inclusion criteria were deliberately more permissive for spray-and-stretch than for ICT in order to represent the modality at all; readers should weight the spray-and-stretch evidence accordingly. Both literatures suffer from difficulty in blinding, heterogeneous outcome definitions, and short follow-up. No quantitative synthesis was performed, and the mechanistic framework in Section 7 is based on indirect reasoning without prospective validation.

The research agenda is concrete. First, a randomised trial directly comparing ICT with the combined spray-and-stretch procedure in a defined myofascial pain population is both feasible and overdue; a three-arm design including a stretch-only comparator would simultaneously test the vapocoolant hypothesis. Second, dismantling designs for spray-and-stretch are needed: spray plus stretch versus sham spray plus stretch versus stretch alone would settle a question open for four decades. Third, the anatomical scope of spray-and-stretch research must widen beyond the upper trapezius. Fourth, ICT trials should routinely report longer follow-up and include an active-exercise-only arm. Fifth, harmonised outcome reporting—a predefined responder threshold, duration of benefit, and pre-specified adverse-event taxonomy—would enable future quantitative synthesis.

## 9. Conclusions

Interferential current therapy and spray-and-stretch address mechanistically distinct aspects of musculoskeletal pain and differ sufficiently in mechanism, evidence base, and practical application that a structured rationale for choosing between them is needed even though no comparative trial yet exists to validate one. ICT is an electrophysical agent applied over a region across multiple sessions, supported by a substantial randomised literature whose consistent message is that its benefit is modest, short-term, and realised mainly as a co-intervention rather than as a stand-alone treatment. Spray-and-stretch is a combined procedure applied to a specific muscle within a single encounter, supported by a much smaller literature demonstrating a real but brief effect of the combined procedure; the independent contribution of the vapocoolant has not been isolated in any dismantling study.

We propose a hypothesis-generating positioning framework—explicitly unvalidated—in which ICT is considered for deep, regional, multi-session analgesic adjunction and the combined spray-and-stretch procedure for single-session restoration of range of motion around a discrete taut band. This framework is a structured set of mechanistic hypotheses to be tested in future comparative trials, not a clinical decision rule. For the research community, the immediate priority is a three-arm trial comparing ICT, the combined spray-and-stretch procedure, and stretch alone, as only such a design can establish whether the vapocoolant contributes anything beyond facilitating stretch, and whether either modality offers value beyond the active treatment it accompanies.

## Figures and Tables

**Table 1:** Mechanistic and technical comparison of ICT and spray-and-stretch.

Feature	Interferential Current Therapy (ICT)	Spray-and-Stretch
Physical agent	Two out-of-phase medium-frequency alternating currents (typically 1–10 kHz)	Volatile topical vapocoolant plus passive manual force applied to the target muscle
Effective stimulus	Low-frequency amplitude-modulated envelope (beat frequency 1–250 Hz)	Abrupt superficial skin cooling followed by passive muscle lengthening
Depth of action	Intended for deep and regional structures; direct in vivo verification limited	Cutaneous cooling only; mechanical effect at the muscle–tendon unit
Proposed mechanism	Segmental gating of Aβ afferents; descending inhibition; endogenous opioid release	Cutaneous counterirritation reducing reflex resistance to stretch (hypothesised)
Unit of intervention	Anatomical region beneath the electrode array	Combined spray-and-stretch procedure applied to a specific muscle and its taut band
Typical dose	20–30 min per session, 3–5 sessions per week, 2–4 weeks	Two to three vapocoolant sweeps with immediate passive lengthening; often one encounter
Vapocoolant independent contribution	Not applicable	Unquantified: no published dismantling study with stretch-only control arm

ICT, interferential current therapy. No statement in this table implies demonstrated efficacy of the vapocoolant independent of the stretch component.

**Table 4:** Hypothesis-generating framework for modality positioning (expert-derived; not prospectively validated).

Clinical Presentation	Research Hypothesis (Not a Clinical Recommendation)	Mechanistic Basis	Key Unresolved Question
Discrete palpable taut band with clear movement restriction in a single accessible muscle	A theoretical basis exists for considering the combined spray-and-stretch procedure	Designed to restore muscle length within one encounter through immediate passive lengthening	Does the vapocoolant contribute beyond facilitating patient tolerance of the stretch?
Diffuse regional pain entering a multi-session rehabilitation programme	A theoretical basis exists for considering ICT as an analgesic co-intervention	Multi-session electrophysical agent; evidence of co-intervention benefit in NSLBP and knee OA	Is the ICT increment maintained beyond the treatment course?
Chronic NSLBP with planned multi-session physiotherapy	ICT as co-intervention may be more applicable; best-supported condition-specific hypothesis	Strongest pooled evidence for ICT (SMD −0.89 vs. sham [22]); incremental benefit vs. exercise alone shown in neck pain trials	Does benefit persist beyond the immediate post-treatment period?
Subacromial or rotator cuff–related shoulder pain	Neither modality is currently hypothesised to offer meaningful benefit	No demonstrated incremental benefit of ICT [28,29]; no evidence for spray-and-stretch in this region	Is progressive tendon loading the appropriate primary treatment?
Post-needling or post-injection soreness with residual range-of-motion restriction	A theoretical basis exists for considering the combined spray-and-stretch procedure	Demonstrated immediate reduction in post-needling soreness in one RCT [34]	Is the effect attributable to the stretch, the spray, or both?
Widespread pain with central sensitisation features	Neither modality is hypothesised to address the dominant mechanism	No discrete peripheral target for either modality	What is the role of graded activity and multidisciplinary care in this phenotype?

This table lists mechanistic hypotheses derived from narrative synthesis and indirect reasoning. No element has been tested in a prospective comparative study, and none constitutes a clinical recommendation. The “deep or inaccessible muscle” row from a previous version of this table has been removed because it rested on the unverified assumption that ICT penetration depth is sufficient to reach deep targets in vivo. NSLBP, non-specific low back pain.

## Data Availability

No new data were created or analysed in this study. Data sharing is not applicable to this article.

## Abbreviations

ICT, interferential current therapy; AMF, amplitude-modulated frequency; MTrP, myofascial trigger point; MPS, myofascial pain syndrome; RCT, randomised controlled trial; TENS, transcutaneous electrical nerve stimulation; WOMAC, Western Ontario and McMaster Universities Osteoarthritis Index; SMD, standardised mean difference; CI, confidence interval; NSLBP, non-specific low back pain; OA, osteoarthritis.
